# Health Information Systems for Older Persons in Select Government Tertiary Hospitals and Health Centers in the Philippines: Cross-sectional Study

**DOI:** 10.2196/29541

**Published:** 2022-02-14

**Authors:** Angely P Garcia, Shelley F De La Vega, Susan P Mercado

**Affiliations:** 1 Institute on Aging National Institutes of Health University of the Philippines Manila Manila Philippines; 2 College of Medicine University of the Philippines Manila Manila Philippines; 3 National Telehealth Center National Institutes of Health University of the Philippines Manila Manila Philippines

**Keywords:** health information systems, the Philippines, aged, hospitals, community health centers, database, geriatric assessment, elderly, digital health, medical records, health policy

## Abstract

**Background:**

The rapid aging of the world’s population requires systems that support health facilities’ provision of integrated care at multiple levels of the health care system. The use of health information systems (HISs) at the point of care has shown positive effects on clinical processes and patient health in several settings of care.

**Objective:**

We sought to describe HISs for older persons (OPs) in select government tertiary hospitals and health centers in the Philippines. Specifically, we aimed to review the existing policies and guidelines related to HISs for OPs in the country, determine the proportion of select government hospitals and health centers with existing health information specific for OPs, and describe the challenges related to HISs in select health facilities.

**Methods:**

We utilized the data derived from the findings of the Focused Interventions for Frail Older Adults Research and Development Project (FITforFrail), a cross-sectional and ethics committee–approved study. A facility-based listing of services and human resources specific to geriatric patients was conducted in purposively sampled 27 tertiary government hospitals identified as geriatric centers and 16 health centers across all regions in the Philippines. We also reviewed the existing policies and guidelines related to HISs for OPs in the country.

**Results:**

Based on the existing guidelines, multiple agencies were involved in the provision of services for OPs, with several records containing health information of OPs. However, there is no existing HIS specific for OPs in the country. Only 14 (52%) of the 27 hospitals and 4 (25%) of the 16 health centers conduct comprehensive geriatric assessment (CGA). All tertiary hospitals and health centers are able to maintain medical records of their patients, and almost all (26/27, 96%) hospitals and all (16/16, 100%) health centers have data on top causes of morbidity and mortality. Meanwhile, the presence of specific disease registries varied per hospitals and health centers. Challenges to HISs include the inability to update databases due to inadequately trained personnel, use of an offline facility–based HIS, an unstable internet connection, and technical issues and nonuniform reporting of categories for age group classification.

**Conclusions:**

Current HISs for OPs are characterized by fragmentation, multiple sources, and inaccessibility. Barriers to achieving appropriate HISs for OPs include the inability to update HISs in hospitals and health centers and a lack of standardization by age group and disease classification. Thus, we recommend a 1-person, 1-record electronic medical record system for OPs and the disaggregation and analysis across demographic and socioeconomic parameters to inform policies and programs that address the complex needs of OPs. CGA as a required routine procedure for all OPs and its integration with the existing HISs in the country are also recommended.

## Introduction

The world’s population is rapidly aging, from the 12% estimate in 2015 to the 22% total global population in 2050 [1]. In the Philippines, 7.5 million, or 7.5%, of the total country population in 2015 were senior citizens (aged 60 years and above) [2]. Recognizing their complex health needs and considering that sound and reliable information is the foundation of decision making across all health systems, the World Health Organization (WHO) developed the Global Strategy and Action Plan on Aging and Health (GSAP 2016-2020), which includes adapting information systems to collect, analyze, and report data on intrinsic trends in the capacity of the aging population [3].

Comprehensive geriatric assessment (CGA) is a form of collecting, analyzing, and reporting data on the intrinsic capacity of an older person (OP). It is a multidimensional, multidisciplinary diagnostic and treatment process conducted by a team of health professionals through a systematic evaluation that identifies a variety of treatable health problems and leads to better health outcomes [4]. It is currently being utilized in different settings, government and private facilities, outpatient and inpatient care, primary care, and research. It contains multiple data points and essential health information about OPs that must be considered in providing holistic and integrated care. Based on findings of meta-analyses [5-10], CGA leads to improved detection and documentation of geriatric problems as well as improvement of health outcomes, such as improvement of functional status, prevention of hospitalization, and reduction in readmission rates or mortality, depending on the specific model and setting in which it is implemented [4]. Furthermore, recent evidence on the cost and effects of CGA showed a reduction in the need for hospital care days in a high-risk population of older adults, which could be of great importance in managing the increasing prevalence of frailty and multimorbidity [11]. This information is also crucial for program and policy development.

One of the main challenges of today’s health system in the country is access to real-time information for decision making [12]. The 2018 Philippine’s health system review highlighted that integrating and harmonizing all existing health-related information systems and data sources, and the inadequacy of a governance structure on information and communication technologies (ICT) are critical challenges [13]. Moreover, the privacy of heath information was also identified as a challenge in policy and practice [14].

The rapid aging of the population requires systems that support health facilities’ provision of integrated care at multiple levels of the health care system. A health information system (HIS) that maintains “1 person, 1 record” facilitates efficient provision of services for OPs. Furthermore, the use of HISs at the point of care has shown positive impacts on clinical processes and patient health in multiple settings of care [15]. The adoption of health information exchange (HIE) programs has proven to lessen utilization of health care services, such as ambulatory care and hospital readmissions, and allow smooth transition from inpatient to outpatient care [16,17].

In recognition of the need for Filipino senior citizens to receive appropriate geriatric health care services, the Department of Health (DOH) provided funding for upgrading the 27 DOH-retained hospitals across regions where geriatric centers will be established [18]. CGA will be conducted in these centers and in primary care settings through Guidelines on the Adoption of Baseline Primary Health Care Guarantees for All Filipinos (DOH Administrative Order [AO] no. 2017-002) [19].

Given the rapid aging population, complex needs of OPs, importance of health information in the delivery of services, and challenges to health information in general, identifying the current status of HISs for OPs is significant in aligning the health system in the country to achieve healthy aging. This is especially true for government tertiary hospitals and health centers where OPs usually access health care.

**Figure 1:**
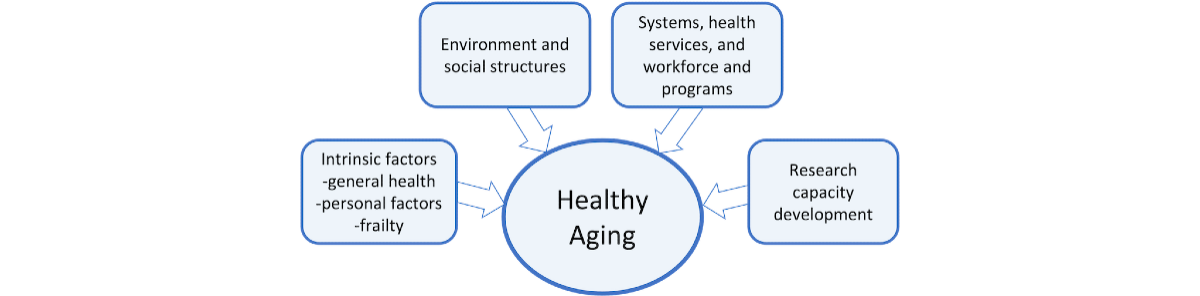
FITforFrail healthy aging framework. FITforFrail: Focused Interventions for Frail Older Adults Research and Development Project.

Figure 1 shows the Focused Interventions for Frail Older Adults Research and Development Project (FITforFrail) framework adapted from the WHO Healthy Aging Framework, which defines healthy aging as the process of developing and maintaining the functional ability that enables well-being in older age [20]. The systems, health services, workforce and programs, intrinsic factors, environment and social structures, and research capacity development are essential parts of the whole-of-system approach that supports healthy aging.

Since healthy aging is the main focus of the GSAP, wherein 1 of the key strategies is aligning health systems to the needs of OPs [3], FITforFrail Study 1 concentrated on the analysis of current health systems for aging in the Philippines. The systems, health services, and workforce and programs, as well as aspects of the environment and social structures, were covered by Study 1, where mixed methods of data collection were utilized.

According to WHO, health systems need to be transformed and realigned to ensure access to evidence-based health interventions responsive to the needs of OPs [3]. A HIS is one of the building blocks of the health system. It provides the underpinnings for decision making where data generation, compilation, analysis and synthesis, and communication and use are its key functions [21]. With the advent of technology, eHealth or the use of ICT for health can maximize its potential toward integrated care of OPs.

The HIS is particularly under the systems, health services, and workforce and programs wherein review of policies related to OPs and listing of services and workforce specific for geriatric patients are conducted.

The data on HISs in general and even among specific population groups in the Philippines are limited. Thus, this paper sought to describe the existing HISs specific for OPs, especially among government tertiary hospitals and health centers across regions in the country. Specifically, it aimed to review the existing policies and guidelines related to HISs for OPs in the Philippines; determine the proportion of select government hospitals and health centers with existing health information specific for OPs, such as CGA, medical records, analysis of top causes of morbidity and mortality, and registry of specific diseases, and electronic medical records (EMRs); and describe the challenges related to HISs in select tertiary hospitals and health centers.

## Methods

### FITForFrail

FITforFrail is a research and development project funded by the DOH through the Philippine Council for Health Research and Development (PCHRD) under the Advancing Health through Evidence-Assisted Decisions with Health Policy Systems Research (AHEAD-HPSR) program. Using the WHO Healthy Aging Framework, the project aims to identify the current health system for the aging population and describe the health status of OPs in select communities. FITforFrail Study 1 analyzed the health system, and FITforFrail Study 2 evaluated the health status of OPs, with a focus on frailty.

### Study Design

A cross-sectional research study design using mixed methods of data collection was utilized. Mixed methods and community participation were hallmarks of this research. For this specific paper, a review of policies and papers related to OPs and a facility-based listing of health services and workforce specific for OPs were conducted to collect the data on HISs.

### Sampling

Purposive sampling was used in selecting study sites. The inclusion criteria for the hospitals were (1) Ministry of Health–/DOH-retained government tertiary hospitals and (2) geriatric centers identified through the Philippine Health Development Plan 2017-2022. For health centers, they had to be within the catchment area of the identified hospital in the region. All the 27 hospitals identified as geriatric centers and 17 health centers within the catchment area across all regions were included in the study.

### Study Setting and Participants

The researchers conducted a listing of health services and workforce specific for OPs in the 27 hospitals identified as geriatric centers and the 17 health centers within their catchment area. An advance copy of the listing tool along with the letter addressed to the heads of the institutions was sent prior to actual data collection. The heads of the institutions assigned and identified focal or point persons to be interviewed to provide their facility data. These identified point persons served as key informants. They were mostly in charge of the geriatric program in their institutions. The research team scheduled separate meetings with the informants to explain the study and obtain consent prior to actual data collection. Policies and the existing literature on HISs were also reviewed.

### Ethics Clearance

FITforFrail obtained a total of 6 ethics approvals from the University of the Philippines Manila Research Ethics Board (UPMREB), the Single Joint Research Ethics Board (SJREB), and 4 institutional review boards of hospitals (Multimedia Appendix 1). The UPMREB oversight applies to UP Manila researchers and non-UP Manila researchers doing research in non-UP Manila sites with no local ethics review committee (as mandated by the Philippine Health Research Ethics Board [PHREB]), while the SJREB is a joint review mechanism among the PHREB–duly accredited research ethics committees (RECs) of DOH hospitals. The rest of the reviews and approvals were from the DOH hospitals that required separate institutional review.

### Data Collection

#### Desk Review

Policies were collected through consultation, online search or bibliographic databases visits, and manual search or onsite library visits. For bibliographic databases and online search, the following search terms were used covering the period of 1980 to July 2020: “aging,” “senior citizens,” “older persons,” “Philippines,” “Republic Act,” “memorandum,” “circulars,” “policy,” “administrative order,” “health information system,” “information systems,” and “programs.”

#### Listing of Services and Workforce

A facility-based listing of services and workforce specific for OPs was conducted. The DOH hospitals identified as geriatric centers were selected as study sites. For primary care units, health centers within the catchment area of the identified regional hospitals were purposely selected across 17 regions in the country. A total of 27 DOH-retained hospitals identified as geriatric centers and 17 health centers were visited for the facility-based listing of services and workforce, with particular attention to HISs for OPs.

A checklist or facility-based listing form was used (Multimedia Appendix 2). The listing form was developed by trained research assistants through policy review and series of consultation meetings with the project and study leaders. The sections of the listing form are as follows: facility demographics, human resources, competencies and training, health services, health financing, information system, and health policies and programs. The specific section on HISs contains questions on patient medical records, disease registries, online databases, and reasons for not having such registries and databases. Moreover, the question on CGA was included under the Health Services section.

### Data Management and Analysis

The collected data from the listing were entered in a password-protected EpiInfo data entry program. Data verification and cleaning were conducted using Microsoft Excel through the help of a statistical assistant and under the supervision of a statistician. Cleaned data sets were endorsed to the statistician for analysis. Descriptive statistics (means, SDs, and frequency distribution) were calculated for all continuous and categorical variables measured using Stata (StataCorp).

## Results

### Policies and Guidelines on Health Information Systems for Older Persons

Republic Act No. 11223 or the Universal Health Care (UHC) Act of 2019 state that all health facilities are required to maintain a HIS consistent with DOH standards, which will be electronically uploaded on a regular basis through interoperable systems [22]. The DOH and PhilHealth will fund and manage the development, quality assurance, and maintenance of the information systems. Under the implementation of the UHC Act is the establishment of a HIS in every health facility, which requires multiple key players for the provision of population- and individual-based health services, including the services for OPs. The DOH, PhilHealth, and the Department of Interior and Local Government (DILG) will integrate all local health systems into a province-wide health system. The private sector will also be encouraged to participate in the integrated local health system through a contractual arrangement.

Prior to the UHC Act, the DOH issued standard policies, procedures, and guidelines governing all ICT-related work in 2005 [13]. It also established the Knowledge Management and Information Technology Service (KMITS); developed the Department of Health Enterprise Architecture (DOH EA) for HISs, which is national in scope; implemented information systems using client-server technology; and established an eHealth framework [23]. A part of the eHealth framework is the Philippine Health Information Exchange (PHIE) through the Joint DOH-DOST-PhilHealth AO no. 2016-001. It aims to achieve integrated health care services and delivery that are also seamlessly responsive, efficient, cost effective, and in real time [24].

The Joint AO no. 2016-003 of DOH and PhilHealth gave way to the adoption of PHIE Lite, which aims to institutionalize the implementation of a harmonized approach and system in developing applications and information systems [25]. OPs were included in the initial priorities of PHIE Lite interoperability as they are included as expanded primary benefit care (ePCB)-entitled sponsored members.

The National Health Data Dictionary (NHDD) and the Unified Health Management Information System (UHMIS), and interoperability standards were also developed and implemented through DOH AO nos. 2013-025 and 2015-037 [26,27]. Unfortunately, the latest version of the NHDD (version 2.0) [28] do not include standard age group classification (young, middle, and oldest old) and relevant diseases, such as geriatric syndromes (ie, dementia, frailty, malnutrition, polypharmacy, and incontinence).

A program dedicated to OPs, the National Health and Wellness Program for Senior Citizens (NHWPSC) through the DOH AO no. 2015-009, was established. One of its objectives is to establish and maintain a database management system and conduct research in the development of evidence-based policies for senior citizens [29]. To date, there is no database management system specific for OPs.

To summarize, multiple agencies are involved in the provision of services for OPs, with several records containing health information about OPs. Moreover, there is no system to integrate or enable interoperability of data systems of OPs at primary, secondary, or tertiary levels of care. Hence, a provider for an OP would be unable to access medical, social, or insurance information in a single record.

### Health Information Specific for Older Persons

Table 1 summarizes the health information for OPs in visited government tertiary hospitals and health centers across all regions in the country.

**Table 1:** Health information for OPs^a^ in government tertiary hospitals and health centers, 2019-2020.

Health information			Hospitals (N=27), n (%)	Health centers (N=16), n (%)
Facilities			27 (100)	16 (100)
CGA^b^			14 (52)	4 (25)
Medical records of patients			27 (100)	16 (100)
Data on top causes of mortality and morbidity			26 (96)	16 (100)
Registry of diseases of OPs			20 (74)	13 (81)
**Diseases in the registry**				
	Hypertension	18 (67)		13 (81)
	Diabetes mellitus	18 (67)		13 (81)
	CVD^c^	18 (67)		10 (62)
	Stroke or cerebrovascular attack	18 (67)		10 (62)
	Heart attack/myocardial infarction	17 (63)		10 (62)
	Respiratory tract diseases	20 (74)		13 (81)
	Cancer	20 (74)		5 (31)
	Mental disorders	7 (26)		3 (19)
	Disability	9 (33)		7 (44)
**Online web-based database**				
	Patient records	23 (85)		10 (62)
	iHoMIS^d^	15 (56)		0
	UDRS^e^	10 (37)		1 (6)
	iClinicSys^f^	0		10 (62)
	Others (Bizbox, MedSys, Medix, CHITS^g^)	8 (30)^h^		1 (6)^i^
	Not updated regularly	4 (15)		6 (37)
**Reasons^j^**				
	No trained/not enough personnel	4 (15)		2 (12)
	Unstable internet	0		2 (12)
	Use of an offline system	2 (7)		1 (6)
	Technical issues	0		2 (12)

^a^OP: older person.

^b^CGA: comprehensive geriatric assessment.

^c^CVD: cardiovascular disease.

^d^iHOMIS: Integrated Hospital Operations and Management Information System.

^e^UDRS: Unified Disease Registry System.

^f^iClinicSys: Integrated Clinic Information System.

^g^CHITS: Community Health Information Tracking System.

^h^Values for Bizbox, MedSys, and Medix.

^i^Value for CHITS.

^j^Multiple responses possible.

### Comprehensive Geriatric Assessment

A total of 27 DOH tertiary hospitals and 17 health centers were visited. Of the 17 health centers, only 16 (94%) have facility-based listing data. There was no information obtained from a health center in Region IV-B, a cluster of islands in southern Luzon, Philippines [30]. The specific question on CGA was in the Health Service Delivery section of the checklist.

The study revealed that only 14 (52%) of the 27 hospitals identified as geriatric centers conduct CGA for their geriatric patients (Table 1). Of these, only 5 (18%) hospitals use CGA to screen for all their geriatric patients; the rest have specific conditions or guidelines regarding to whom they can administer CGA. Most hospitals would only utilize CGA in specific age brackets; other hospitals would only do so through referrals, when the patient is admitted, or when they think the patient is frail or at risk. Commonly reported reasons for not administering CGA to all OPs in hospitals include the lack of manpower, inadequate trained personnel, and the length of the assessment. However, of the 16 health centers, only 4 (25%) conduct CGA for their geriatric patients.

### Medical Records and Registries for OPs in Hospitals and Health Centers

All 27 hospitals and 16 health centers maintain medical records of their patients. The data on the top causes of mortality are available in almost all (26/27, 96%) visited hospitals and all (16/16, 100%) health centers. When asked whether the facilities have a registry of diseases of OPs, there are more health centers than hospitals that have these (13/16 [81%] vs 20/27 [74%]), as summarized in Table 1.

In terms of specific registries (Table 1), hospitals have better registries on cardiovascular disease (CVD; 18/27 [67%] vs 10/16 [62%]), stroke (18/27 [67%] vs 10/16 [62%]), heart attack (17/27 [63%] vs 10/16 [62%]), cancer (20/27 [74%] vs 5/16 [31%]), and mental disorders (7/27 [26%] vs 3/16 [19%]). However, health centers have better registries on hypertension (13/16 [81%] vs 18/27 [67%]), diabetes (13/16 [81%] vs 18/27 [67%]), respiratory tract diseases (13/16 [81%] vs 20/27 [74%]), and disability (7/16 [44%] vs 9/27 [33%]). Whether these registries are or are not CGA based is not known, as this was not covered by the study and was considered 1 of its limitations.

There are more hospitals that utilize online web-based database of patients records than health centers (23/27 [85%] vs 10/16 [62%]). More than half (15/27, 56%) of the hospitals utilize the Integrated Hospital Operations and Management Information (iHOMIS), and more than a quarter (10/27, 37%) utilize the Unified Disease Registry System (UDRS). iHOMIS is a Windows-based computerized hospital information system for government hospitals, while the UDRS is a unified registry that contains an injury surveillance system, an integrated noncommunicable diseases registry, a violence against children and women registry, and a persons with disabilities registry [31].

Other third-party providers, such as BizBox, MedSys, and Medix, were also reported. Bizbox is a PhilHealth-accredited health information technology provider that passed the eClaims certification on the case rate system [32]. The MedSys EMR is a web-based application developed for physicians and staff within a health care institution to ensure accuracy, privacy, and service efficiency [33]. Lastly, Medix is a cloud-based clinic management software that helps practitioners improve their clinic operations [34]. Some of these are also being utilized by government hospitals despite the availability of DOH-maintained iHOMIS.

For the health centers, more than half (10/16, 62%) utilize an online web-based database for patient records through the Integrated Clinic Information System (iClinicSys), while only 1 (6%) uses the Community Health Information Tracking System (CHITS), as shown in Table 1. iClinicSys is a system owned by the DOH that efficiently and effectively monitors patient cases in rural health units (RHUs) [31], while CHITS is an EMR system for government primary care health centers in the Philippines [35].

### Challenges Related to HISs in Select Tertiary Hospitals and Health Centers

In terms of management of HISs, the most common reasons for not regularly updating the web-based database are a lack of or inadequate trained personnel to maintain and manage the information systems (in 4/27 [15%] of hospitals and 2/16 [12%] of health centers), an unstable internet connection (2/16 [12%] of health centers), the use of an offline system (1/16 [6%] of health centers), and technical issues (2/16 [12%] of health centers), as shown in Table 1.

## Discussion

### Principal Findings

This study described HISs specific for OPs, especially among government tertiary hospitals and health centers across regions in the Philippines. It reviewed the existing policies and guidelines and determined the proportion of select government hospitals and health centers with existing health information specific for OPs, such as CGA, medical records, top causes of morbidity and mortality, registries of specific diseases, and EMRs. Furthermore, challenges related to HISs in select health facilities were described.

There are various HISs in the country. For primary care benefit providers, the following are the DOH-accredited EMR systems: iClinicSys, CHITS, Segworks Tecknologies (Seg-RHIS), the eHatid local government unit (LGU), Secure Health Information Network and Exchange (SHINE OS+), and Wireless Access for Health (WAH) [36]. Furthermore, the DOH maintains 10 information systems and databases. These include the Electronic Drug Price Monitoring System (EDPMS), iClinicSys, the Integrated Chronic Non-Communicable Disease Registry System (ICNCDRS), the Integrated Drug Test Operations Management Information System (IDTOMIS), iHOMIS, the Integrated TB Information System (ITIS), the Online National Electronic Injury Surveillance System (ONEISS), the Philippine Registry for Persons with Disabilities (PRPWD), the National Rabies Information System (NaRIS), and the Violence Against Women and Children Registry System (VAWCRS) [31]. In addition, there are other private or third-party providers of HISs in the country, such as BizBox, MedSys, and Medix.

Among the existing HISs maintained by the DOH, there is no specific one for OPs. The data on OPs can be distributed in almost all existing HISs (ie, PRPWD; ICNCDRS; online reporting of cancer, diabetes, chronic obstructive pulmonary disease, stroke, blindness, mental, coronary artery disease, and renal data from health facilities; ITIS; ONEISS; and other HISs). All these systems require log-in credentials; thus, only authorized personnel have access.

Based on the policies and literature review, there are policies and guidelines that support the establishment and integration of HISs for OPs. However, there is no current database management system specific for OPs to date, and the data from the existing HISs maintained by the DOH are not readily accessible. Geriatric syndromes, including frailty, malnutrition, dementia, incontinence, and polypharmacy, are not in the NHDD.

There are multiple information systems and agencies involved in the provision of services and sources of health information about OPs, which leads to fragmented health information about OPs in the country. Given the limited accessibility and fragmentation, coming up with evidence for program and policy development that will address the needs of OPs is a major challenge.

More than half of the hospitals identified as geriatric centers and only a quarter of the health centers conduct CGA for their geriatric patients. According to the DOH AO no. 2017-001, “All older patients with a positive risk screen should have a Comprehensive Interdisciplinary Geriatric Assessment for individual special complex needs” and the “Comprehensive Geriatric Assessment should be updated prior to discharge in chronic care facilities and made available to accepting facilities or carers and vice versa” [37].

This study found a limitation in the conduct of CGA, especially in the primary care setting. Not all visited DOH hospitals, although being identified as geriatric centers, conduct CGA. The commonly reported reasons for not administering CGA to all OPs in hospitals include the lack of personnel, inadequate trained personnel, and the length of the assessment.

All visited hospitals and health centers maintain medical records of their patients. The data on the top causes of mortality are available in all health centers and almost all visited hospitals. There are more hospitals that utilize online web-based databases of patients records than health centers. More than half of the hospitals utilize iHOMIS, and more than a quarter utilize the UDRS. In addition, there are third-party providers, such as BizBox, MedSys, and Medix.

There are more health centers than hospitals that have a specific registry of diseases. Hospitals have better registries on CVD, stroke, heart attack, cancer, and mental disorders. However, health centers have better registries on hypertension, diabetes, respiratory tract diseases, and disability. More than half of the health centers visited utilize an online web-based database for patient records through iClinicSys, while only 1 uses another information system, specifically CHITS.

Most of the information systems utilized by the hospitals and health centers are for all patients in general wherein data on OPs can only be extracted. However, the extraction of data on OPs is complicated due to the nonuniform age group categories. In some facilities, the data on patients aged 60-64 years could not be properly retrieved, as these are incorporated into the 45-64-year age group. Age group classification is not standardized across facilities. Having multiple platforms for managing health information deteriorates interoperability between different health facilities, which, in effect, reduces the ease of service delivery.

### Limitations

The study was able to cover facilities representing each region across the country; however, these are limited to the selected hospitals identified as geriatric centers and the health centers within their catchment area. Private health facilities were not covered by the study. Thus, the status of HISs in this study was limited only to public health facilities. Moreover, the status of the Philippines’ HISs in general was not within the scope the study and thus warrants further investigation.

### Comparison With Prior Work

In 1990, the BLACKBOX was the management information system for public health programs, vital statistics, mortality, and notifiable diseases. It handled and retrieved all data that were being routinely collected by public health workers all over the Philippines. It was developed toward a need-responsive and cost-effective health and management information system (HAMIS) [38]. Decades later, with the advancement of eHealth, there are various HISs in the country. For primary care benefit providers, there are 6 DOH-accredited EMR systems [36]. Furthermore, the DOH maintains 10 information systems and databases, which are being implemented in various health care settings through the UHMIS [31]. In addition, there are other private or third-party providers of HISs in the country. These are harmonized through the interoperability standards and guidelines issued by the DOH. However, based on the results of this study, there is no current database management and HIS specific for OPs to date.

The National Objectives for Health 2005-2010 and 2011-2016 prioritized the use of ICT in various reforms areas, critical health programs, and specific areas in health administration [39,40]. The Philippine eHealth Strategic Framework and Plan 2014-2020 was also developed [23]. The current and overall status of the PHIE warrants further investigation.

In terms of management of HISs, the most common reasons for not regularly updating the web-based database are a lack of or inadequate trained personnel to maintain and manage the information system, an unstable internet connection, the use of an offline system, and technical issues. These barriers are also consistent with the findings of other studies, such as a lack of standards, the use of different information systems, infrastructure issues for electricity and connectivity [35], a lack of human expertise [41], the need for training and support for human resources [41,42], and technical complexity [43,44]. In Malaysia, several issues have influenced overall HIS implementation in public hospitals, such as limited financial sources, maintenance by different departments, HIS implementation orders by the Malaysian Ministry of Health, addition of new systems, confidentiality issues, low acceptance levels, low satisfaction levels, different vendors, infrastructure issues, system breakdown, duplication of data, and different systems [45].

In developing countries, the establishment of well-coordinated information collection systems at various levels of the health care system using appropriate staff could contribute greatly to improvements in health care delivery [46]. Furthermore, ICT need to be seen as part of wider approaches involving technological, social, and institutional innovation; health workers need to be educated more broadly on the use of HISs for action [46,47]; health institutions need to adapt in many ways toward local accountability and patient and health worker empowerment; and software development for HISs needs to integrate computerized systems with work practices to make work more effective [47]. In decentralized and democratic governments similar to the Philippines, HISs can play a crucial role in supporting and sustaining processes by serving as a repository for generated and analyzed information at the local level so that primary health care can address the dynamic and unpredictable elements of health care planning in developing countries [48].

Routine HIS interventions in the European region were identified to be promising; however, different areas of improvement, such as technical, organizational, and behavioral elements, were identified [49]. In Japan, the areas of improvement in health care information technology include the necessity for leadership and IT knowledge in medical communities, provider incentives, legislation regarding accountability, security, privacy and confidentiality, inclusion of stakeholders in solution development, and creation of sustainable business models [50].

In terms of sustainability of HISs, many challenges are faced, and these could be addressed through the systems’ technical design, stakeholder coordination, and the building of organizational capacity to maintain and enhance such systems [51]. Furthermore, effective collaboration between major actors (donors, developers, and the Ministry of Health) is fundamental to sustain HISs [52].

### Conclusion and Recommendations

The review of existing policies and guidelines provided a background on the status of HISs for OPs in the Philippines. The facility-based listing revealed the proportion of select facilities that conduct CGA and the status and challenges related to the HIS in select tertiary hospitals and health centers in the country.

Current HISs for OPs are characterized by fragmentation, multiple sources of health information, and inaccessibility. Barriers to achieving appropriate HISs for OPs include inability to update HISs in hospitals and health centers and a lack of age group and disease standardization.

A comprehensive assessment and care plan shared with all providers is one of the important elements of integrated care for OPs. In line with the universal health coverage and Sustainable Development Goal of “*Ensuring healthy lives and promote wellbeing for all at all ages*,” an emerging landscape of innovation and development on integrated care of OPs is essential in order to address the multidimensional needs of the aging population.

A 1-person, 1-record EMR system for OPs is recommended in order to address their complex needs, as well as extract data to inform policies and programs. Furthermore, the data on OPs should be disaggregated and analyzed across geographic and social parameters in order to identify gaps in programs and provision of services.

Specifically, we recommend the following:

- Integration of data of OPs in the existing HISs in the country, wherein data can be derived and disaggregated across all health care facilities
  - Standardizing the definition of age groups (young, middle, and oldest old) and geriatric syndromes (ie, frailty, malnutrition, falls, dementia, delirium, incontinence, polypharmacy, deconditioning) and inclusion in the latest version of the NHDD (KMITS-DOH)
  - Funding and creating a dashboard for OPs (DOH, PhilHealth)
  - Conducting a CGA of all OPs as a clinical record to be shared across health care providers in all health settings, which will be integrated in the existing HIS
- Alignment of the integration of HISs for OPs with the existing mandates of the NHWPSC and health care provider networks (NHWPSC-DOH, centers for health development [CHDs], LGUs)
- Hiring and capacity building of personnel for management and maintenance of facility-based HISs (Health Human Resource Development Bureau [HHRDB]-DOH, regional hospitals, LGUs)
- Research, evaluation, and monitoring of the integrated HIS (National Commission of Senior Citizens [NCSC], National Privacy Commission [NPC], Health Policy Development and Planning Bureau [HPDPB]-DOH, academia, research institutions)

## Appendix

Multimedia Appendix 1 Ethics approval compilation.

Multimedia Appendix 2 Checklist for hospitals and health centers.

## Abbreviations

AO: Administrative Order
CHITS: Community Health Information Tracking System.
CGA: comprehensive geriatric assessment
DILG: Department of Interior and Local Government
DOH: Department of Health
DOH EA: Department of Health Enterprise Architecture
EDPMS: Electronic Drug Price Monitoring System
EMR: electronic medical record
ePCB: expanded primary benefit care
FITforFrail: Focused Interventions for Frail Older Adults Research and Development Project
GSAP: Global Strategy and Action Plan on Aging and Health
HAMIS: health and management information system
HIE: health information exchange
HIS: health information system
iClinicSys: Integrated Clinic Information System.
ICNCDRS: Integrated Chronic Non-Communicable Disease Registry System
ICT: information and communication technologies
IDTOMIS: Integrated Drug Test Operations Management Information System
iHOMIS: Integrated Hospital Operations and Management Information System.
ITIS: Integrated TB Information System
KMITS: Knowledge Management and Information Technology Service
LGU: local government unit
NaRIS: National Rabies Information System
NHDD: National Health Data Dictionary
NHWPSC: National Health and Wellness Program for Senior Citizens
ONEISS: Online National Electronic Injury Surveillance System
OP: older person
PCHRD: Philippine Council for Health Research and Development
PHIE: Philippine Health Information Exchange
PHREB: Philippine Health Research Ethics Board
PRPWD: Philippine Registry for Persons with Disabilities
REC: research ethics committee
RHU: rural health unit
Seg-RHIS: Segworks Tecknologies
SHINE OS+: Secure Health Information Network and Exchange
SJREB: Single Joint Research Ethics Board
UDRS: Unified Disease Registry System.
UHC: universal health care
UHMIS: Unified Health Management Information System
UPMREB: University of the Philippines Manila Research Ethics Board
VAWCRS: Violence Against Women and Children Registry System
WAH: Wireless Access for Health
WHO: World Health Organization
