# Supplementary material for: Health Information Systems for Older Persons in Select Government Tertiary Hospitals and Health Centers in the Philippines: Cross-sectional Study
Source: J Med Internet Res. 2022 Feb 14;24(2):e29541. doi: 10.2196/29541 (PMC8887638; doi:10.2196/29541)
Supplement: Multimedia Appendix 1 [file jmir_v24i2e29541_app1.pdf]

University of the Philippines Manila
RESEARCH ETHICS BOARD
Room 126 NIH Building, University of the Philippines Manila
547 Pedro Gil Street, Ermita, 1000 Manila
Telephone: +63 2 5222684; *Mobile*: +63 927 3264910; *Email*: upmreb@post.upm.edu.ph

22 February 2019

DR. SHELLEY ANN DE LA VEGA
Principal Investigator
Institute on Aging
National Institutes of Health - UP Manila

Re: UPMREB 2017-422-01
Project 2 Focused InTerventions for FRAIL Older Adults Research and Development Program: (FITforFRAIL)

Dear **Dr. de la Vega**:

We wish to inform you that the UP Manila Research Ethics Board (UPMREB) Review Panel 2 acknowledged receipt of Continuing Review Application dated 13 December 2018.

Upon review of UPMREB FORM 3(B)2012: Continuing Review Application Form Panel action is **APPROVED**. Ethical clearance is valid until **29 February 2020**. The report is noted and has been included in the protocol file.

Very truly yours,

DR. VIRGINIA DE JESUS
Chair, UPMREB Review Panel 2

CERTIFICATION OF APPROVAL

This certifies that the **University of the Philippines Manila Research Ethics Board (UPMREB) Review Panel 2** which is constituted and established, and functions in accordance with the requirements set by the University of the Philippines Manila, the Philippine Health Research Ethics Board (PHREB); and in compliance with the WHO Standards and Operational Guidance for Ethics Review of Health-related Research with Human Participants (2011), the International Council for Harmonisation of Technical Requirements for Pharmaceuticals for Human Use (2016), and the National Ethical Guidelines for Health and Health-related Research (2017), has approved the following study protocol and related documents:

**TYPE OF SUBMISSION:** Continuing Review	
**UPMREB CODE:** 2017-422-01	
**SUBMISSION DATE:** 20 March 2020	
**STUDY PROTOCOL TITLE:** Project 2 Focused InTerventions for FRAIL Older Adults Research and Development Program: (FITforFRAIL)	
**PRINCIPAL INVESTIGATOR:** Dr. Shelley Ann de la Vega	
**TYPE OF REVIEW:** Full Board	
**SPONSOR/FUNDING AGENCY:** AHEAD HPSR DOH	
**APPROVAL DATE:** 21 April 2020	**EXPIRY OF ETHICAL CLEARANCE*:** 30 April 2021
**DUE DATE OF APPLICATION FOR RENEWAL OF ETHICAL CLEARANCE (30 days before expiration date):** 31 March 2021	**FREQUENCY OF CONTINUING REVIEW:** Yearly
**APPROVED SITES:** Institute on Aging, NIH UP Manila	
**DATE OF BOARD MEETING:** 21 April 2020	
**QUORUM: Quorum present**	
**CONFLICT OF INTEREST: None declared**	
**MEMBERS IN ATTENDANCE:**<br>Dr. Virginia De Jesus (Chair)<br>Dr. Patricia Khu (Secretary)<br>Dr. Cecilia Conaco<br>Dr. Noel Juban<br>Prof. Edwin Ruamero, Jr.<br>Dr. Josephine Agapito<br>Mrs. Agnes Vizco<br>Mrs. Rosalita Tan	ALTERNATE MEMBERS<br>Dr. Leslie Michelle Dalmacio<br>Mrs. Felicidad Romualdez<br>Dr. Cherica Tee
**ACTION:** Approved	

RESPONSIBILITIES OF PRINCIPAL INVESTIGATOR WHILE STUDY IS IN PROGRESS
(*Please note that forms may be downloaded from the UPMREB website: **reb.upm.edu.ph***):

1. Register research study in the Philippine Health Research Registry upon approval (http://registry.healthresearch.ph)
2. Progress report using the attached UPMREB FORM3(B)2012: Continuing Review Application Form, as indicated above, which includes the following: *(NOTE: In view of active ethical clearance, this report is mandatory even if the study has not started or is still awaiting release of funds.)*
    a. Date covered by the report
    b. Protocol summary and status report on the progress of the research
    c. Philippine Health Research Registry ID
    d. Number of participants accrued
    e. Withdrawal or termination of participants
    f. Complaints on the research since the last UPMREB review
    g. Summary of relevant recent research literature, interim findings and amendments since the last UPMREB review
    h. Any relevant multi-center research reports
    i. Any relevant information especially about risks associated with the research
    j. A copy of the informed consent document
3. Any amendment/s in the protocol, especially those that may adversely affect the safety of the participants during the conduct of the trial including changes in personnel, and revisions in the informed consent, must be submitted or reported using UPMREB FORM3(A)2012: Study Protocol Amendment Submission Form.
4. Report of non-compliance (deviation/violation), whether minor or major, at the soonest possible time up to six (6) months after the event, using UPMREB FORM 3(D)2012: Study Protocol Non-Compliance (Deviation/Violation) Report.
5. Reports of adverse events including from other study sites (national, international) using the UPMREB FORM 3(G)2012: Suspected, unexpected serious adverse event/reaction/s report, with timelines for submission guided by the GL 02 Version 2.0: Guideline on Reporting Serious Adverse Events; or list of reportable negative events using the UPMREB FORM 3(I)2012: Queries, Notification, and Complaints.
6. Notice of early termination of the study and reasons for such using UPMREB FORM 3(E)2012, or notice of time of completion of the study using UPMREB FORM 3(C)2012: Final Report Form.
7. Any event which may have ethical significance, and/or any information which is needed by the UPMREB to do ongoing review.

DR. VIRGINIA DE JESUS
Chair, UPMREB Review Panel 2

Republic of the Philippines
Department of Health
SINGLE JOINT RESEARCH ETHICS BOARD

SJREB FORM 5
NOTICE OF APPROVAL

Date: April 29, 2019

This is to certify that the following protocol and related documents have been granted approval by the SJREB for implementation in accordance with the International Conference on the Harmonization of Good Clinical Practice and the National Ethical Guidelines on Health and Health-related Research

SJREB Protocol No.:	SJREB-2019-01	Sponsor Protocol No.:	N/A
Coordinating Investigator:	Dr. Shelly de la Vega	Sponsor:	DOH – AHEAD HPSR
Title:	Focused Interventions for Frail Older Adults Research and Development Program		
Protocol Version No.:	V1	Version Date:	12 March 2019
ICF Version No.:	FGD ICF – V1<br>KII ICF – V1<br>Survey ICF – V1	Version Date:	05 March 2019
Other Documents:			
Members of research team:	Dr. Shelly de la Vega, Dr. Mary Ann Ladia, Dr. Maria Stella Giron, Ms. Angely Garcia, Ms. Hannah Pellejo, Dr. Nimfa Ogena, Dr. Grace Cruz		
Study sites:	Cebu and Davao		
Type of Review:	[ ] Expedited<br>[X] Full Board<br>Meeting date: February 13, 2019	Duration of Approval From – To *(date)*<br>April 29, 2019 to April 19, 2020	Frequency of continuing review<br>Annual

- **Approval with the condition that the team submits the developed questionnaire once available**

SJREB Chair	Signature	Date
Dr. Jacinto Blas Mantaring III	[signature]	

Investigator Responsibilities after Approval:

- Submit document amendments to the site REC approval before implementing them;
- Submit annual report for renewal of approval to SJREB;
- Submit SAE and SUSAR reports to the site REC within 7 days;
- Submit progress report every 12 months;
- Submit final report after completion of protocol procedures at the study site;
- Report protocol deviation/violation to the REC study sites;
- Comply with all relevant international and national guidelines and regulations; and
- Abide by the principles of good clinical practice and ethical research

Received by:

Name: [signature]

Signature: Angely P. Garcia

Date: 04/29/19

Republic of the Philippines
Department of Health
'AMANG' RODRIGUEZ MEMORIAL MEDICAL CENTER
Marikina City
"PHIC Accredited"

ETHICS REVIEW BOARD
APPROVAL LETTER FOR INITIAL REVIEW (Comm. Form 2.02)

October 2, 2019

TO: **SHELLEY ANN F. DE LA VEGA, MD, MSc**
Principal Investigator

RE: **PROTOCOL EVALUATION**

Dear Dr. de la Vega,

We wish to inform you that the ARMMC-ERB has reviewed your protocol and has granted its approval for implementation, to wit:

ARMMC-ERB Protocol No:	**2019-03-00**
Sponsor Protocol No:	**N/A**
Research/Project Title:	**Focused InTerventions for FRAIL Older Adults Research and Development Program: (FITforFRAIL), Study 1: Analysis of Health Systems on Aging**
Approval Date :	**October 1, 2019**
Expiration Date:	**September 30, 2020**
Type of Approval :	☑ Expedited ☐ Full Review

Please be reminded that after this approval, the following are your responsibilities:

1. This approval expires on the date indicated above. For renewal, submit to us a Progress Report every year (use *Form 3.05*);
2. Inform the ERB of any modifications or changes in the research design which affect human participants, or changes in consent documents, prior to implementation (use *Form 3.03)*;
3. Report to us any protocol deviation / violation (use *Form 3.07*);
4. Submit SAE *(serious adverse event)* and SUSAR *(suspected unexpected serious adverse reaction)* reports within seven days of the occurrence of the event (use *Form 3.02*);
5. Upon completion of the study, inform us in writing by submitting a Final Accomplishment Report (*Form 3.06*);
6. You are to comply with all international and national guidelines and regulations, and abide by the principles of good clinical practice and ethical research.

For your information and guidance.

for
THEODOR S. VESAGAS, MD, FPCS, FAFN
Chair, ARMMC-ERB

Republic of the Philippines
Department of Health
'AMANG' RODRIGUEZ MEMORIAL MEDICAL CENTER
Marikina City
"PHIC Accredited"

ETHICS REVIEW BOARD
ACTION DECISION (Form 2.08)

ARMMC-ERB Control No:	2019-03-00
Sponsor Protocol No:	N/A
Review Date:	October 1, 2019

Protocol Title:	**Focused InTerventions for FRAIL Older Adults Research and Development Program: (FITforFRAIL), Study 1: Analysis of Health Systems on Aging**

Type of Submission:

- [x] Initial Review
- [ ] Continuing Review
- [ ] Resubmission for re-review
- [ ] Protocol Termination
- [ ] Protocol Amendment(s)
- [ ] Final Report

Type of Review:

- [x] Expedited
- [ ] Full Board Review
- [ ] SJREB
- [ ] Exempt

ARMMC-ERB Decision

- [x] Approved
- [ ] Minor revisions required
- [ ] Major revisions required
- [ ] More information required
- [ ] Others

Name of Chair	Signature
FOR THEODOR S. VESAGAS, MD, FPCS, FAFN	[signature]

Date
October 2, 2019

RIZAL MEDICAL CENTER
DEPARTMENT OF HEALTH
Pasig Blvd., Pasig City, Philippines info@rmc.doh.gov.ph (02) 865-84-00

October 3, 2019

SHELLEY ANN F. DELA VEGA, MD, MSC
Project Leader, FITforFRAIL
Professor, UP College of Medicine
Director, Institute on Aging NIH
University of the Philippines Manila

Dear **DR. DELA VEGA**;

This is to inform you that your project proposal entitled " Project 2 Focused Interventions for FRAIL Older Adults Research and Development Program (FITforFRAIL) has been already **APPROVED** and has been assigned the following Protocol Registration Code ***2019-Z-#105-RP-BOARD.I*** which shall expire on ***10 October 2020*** unless extension is requested.

The following are the recommendations of the RizalMed Institutional Review Board:

1. There will be no separate review of the protocol.
2. The Approval of the UP Research Ethics Board shall be honored.
3. A study coordinator for RizalMed shall be assigned to liaise between the members of the research team and the various offices and personalities that shall be involved in the study.
4. The Board is recommending **Dr. Niña Teresa S. Bravo**, Medical Specialist II and Research Coordinator for the Department of Internal Medicine to be the study coordinator.
   Please coordinate with her through her email ninasisonbravo@gmail.com
5. There will be no institutional fee charged for the study since it also a government funded study, but we suggest that an honoraria be given to the Study Coordinator.
6. The copy of the final manuscript shall be given to the RizalMed IRB for archiving.

Respectfully yours,

CARMELA B. ENCARNACION, MD, FPPA
Chair, Rizal Med-Institutional Review Board

	Republic of the Philippines<br>Department of Health, Center for Health Development (CHD) IV-CALABARZON<br>**BATANGAS MEDICAL CENTER**<br>Batangas City<br>**ISO 9001:2015 CERTIFIED**	

APPROVAL LETTER

Date: December 10, 2019

This is to certify that the following protocol and related documents have been granted approval by the Batangas Medical Center RERC for implementation

RERC Protocol No.	BATMC-RERC-2017-033	Sponsor Protocol No.	N/A
Principal Investigator/s	Shelly Ann F. De la Vega,MD	Sponsor	
Title	Focused Interventions for Frail Older Adults Research and Development Program (FITforFRAIL)Study 1: Analysis of Health System on Aging		
Protocol Version No.	BatMC-RERC-2017-033 v.1	Version Date	November 22, 2019
ICF Version No.	v.1	Version Date	November 22, 2019
Other Documents	None		
Type of Review	✓Expedited<br>☐Full Board	Duration of Ethical Clearance<br>December 10, 2019 to December 10, 2020	Submission of Progress Report:<br>March 10, 2020

**RERC-Chairman**	**Name**<br>**RHODORA M. REYES, MD**	**Signature**<br>[signature]	**Date**<br>**12/10/2019**

Investigator Responsibilities after Approval:

- Submit document amendments for RERC approval before implementing them
- Submit SAE and SUSAR reports to the RERC within 7 days
- Submit progress report every 3 months.
- Submit final report after completion of protocol procedures at the study site
- Report protocol deviation/ violation
- Comply with all relevant international and national guidelines and regulations
- Abide by the principles of good clinical practice and ethical research

Received by:

SHELLEY ANN F. DE LA VEGA MD, MSc
PROJECT LEADER, FITFORFRAIL
Signature over Printed Name

Date: 12/12/19

Excerpt from DOH Standard Operating Procedures
for Hospital Ethics Review Committee, Year 2017, (Form 2.7)

QUIRINO MEMORIAL MEDICAL CENTER
RESEARCH ETHICS BOARD
Form 2.10 CERTIFICATE OF APPROVAL

Date 06 November 2019

This is to certify that the following protocol and related documents have been granted approval by the Quirino Memorial Medical Center REB for implementation

REB Protocol No.	QMMC REC GCS 2019-83	Sponsor Protocol No	NA
Principal Investigator/s	SHELLEY ANN F. DELA VEGA, MD	Sponsor	DOH – AHEAD HPSR
Title	FOCUSED INTERVENTIONS FOR FRAIL ORDER ADULTS RESEARCH AND DEVELOPMENT PROJECT (FIT FOR FRAIL)		
Protocol Version No.	VI	Version Date	12 MARCH 2019
ICF Version No.	FGD ICF – VI<br>KII ICF – VI<br>SURVEY ICF - VI	Version Date	05 MARCH 2019
Other Documents	COVER LETTER TO QMMC REB DATED 02 OCTOBER 2019<br>QMMC REB FORMS 2.1, 2.2, 2.3A, 2.4, 2.5<br>CURRICULUM VITAE AND GCP CERTIFICATE OF THE PRINCIPAL INVESTIGATOR		
Members of research team	SHELLEY ANN F. DELA VEGA, MD<br>MARY ANN LADIA, MD<br>MARIA STELLA GIRON, MD<br>ANGELY GARCIA, MD<br>HANNAH PELLEJO, MD<br>NIMFA OGENA, MD<br>GRACE CRUZ, MD		
Study site	QUIRINO MEMORIAL MEDICAL CENTER		

Type of review		Duration of Approval	Frequency of Progress Reports	Frequency of Continuing Review
	■ Expedited<br>☐ Full board	From: 06 NOVEMBER 2019<br>To: 06 NOVEMBER 2020	Every 12 months	Annual

Investigator Responsibilities after Approval:

- Submit document amendments for REB approval before implementing them
  (Form 3.1 Protocol Amendment Application)
- Submit SAE and SUSAR reports to the REB within 7 days
  (Form 3.4 Onsite Serious Adverse Event Report)
- Report any protocol deviation/violation
  (Form 3.5 Protocol Violation-Deviation Report)
- Submit progress report every 6 months
  (Form 3.2 Continuing Review and Progress Report)
- Application for renewal of ethical clearance should be thirty (30) days before the approval expiration date
  (Form 3.2 Continuing Review and Progress Report)
- Submit final report after completion of protocol procedures at the study site
  (Form 3.3 Closure/ Final Report)
- Comply with all relevant international and national guidelines and regulations
- Abide by the principles of good clinical practice and ethical research

The Committee **APPROVES** the implementation of the research protocol. Please be guided by the policies, rules and regulation of the Quirino Memorial Medical Center pertaining the conduct of industry-sponsored research as well as of the QMMC Research Ethics Board.

Furthermore, we would like to inform you that the Quirino Memorial Medical Center Research Ethics Board is organized and operates according to Good Clinical Practice and applicable laws and regulations.

Please note that QMMC REB Forms may be requested through *qmmc.ethics@gmail.com.*

Very truly yours,

MA. FREDELITA C. ASUNCION, MD, FPCP, FPSAAI

Chairman, Research Ethics Board
Quirino Memorial Medical Center

Date: 06 November 2019
