# Supplementary material for: Health Information Systems for Older Persons in Select Government Tertiary Hospitals and Health Centers in the Philippines: Cross-sectional Study
Source: J Med Internet Res. 2022 Feb 14;24(2):e29541. doi: 10.2196/29541 (PMC8887638; doi:10.2196/29541)
Supplement: Multimedia Appendix 2 [file jmir_v24i2e29541_app2.pdf]

INSTITUTE ON AGING
National Institutes of Health

FITforFRAIL

Review of Health Services

HOSPITAL

Name of Facility: ____

I. Facility Demographics

I.A. Hospital Classification

I.A.1. Level 1	
I.A.2. Level 2	
1.A.3 Level 3	

I.B. Do you have a Geriatric care program in your facility?

I.B.1. Yes ◯   1.B.2. No ◯

I.C. Type of Geriatric Program in the Facility

	I.C.1. Geriatric Center
	I.C.2. Geriatric Department
	I.C.3. Geriatric Ward
	I.C.4. Out-patient Geriatric Services
	I.C.5. Others (please specify)

I.D. Who manages the Geriatric Program?

	I.D.1. Geriatric Department
	I.D.2. Internal Medicine Department
	I.D.3. Family Medicine Department
	I.D.4. Out-patient Department
	I.D.5. Chief of Hospital
	I.D.6. Others *(please specify):*

CONFIDENTIAL

I.E. What is your mechanism in monitoring the performance of the geriatric program? *(check all that applies)*

	I.E.1 Annual program implementation review
	I.E.2. Self-rated performance evaluation review
	I.E.3. Peer-rated performance evaluation review
	I.E.4. Supervisor-rated performance evaluation review
	I.E.5. Patient feedback surveys
	I.E.6. Admission - Discharge - Re-admission rate
	I.E.7. Special Audit
	I.E.8. Regular Meetings
	I.E.9. Others *(please specify)*

I.F. Total Bed Capacity:	
I.G. Total number of beds allotted for geriatric care:	
I.H. Total number of patients (2018):	
I.I. Total number of patients age 60 years old and above (2018):	
I.J. Total number of admissions (2018):	
I.K. Total number of admissions of 60 year old and above (2018)	
I.M. Total number of out-patients (2018):	
I.N. Total number of 60 years old and above out-patient consultations (2018):	

CONFIDENTIAL

II. Human Resource, Competencies and Training

Workforce	Total Number	Competencies and Trainings			
		Formal (*specialization or post graduate degree in geriatrics or gerontology*)		Informal (*Certificate of Training in the Conduct of Geriatric Assessment and Management*)	Trainings (*Other trainings related to the care of the Senior Citizens*)
		Completed	On-going		
II.A. Doctor of Medicine					
II.A.1. Specialist					
II.A.1.a. Geriatrician					
II.A.1.a.i. permanent					
II.A.1.a.ii. non-permanent					
II.A.1. Others (please specify / follow format above – specialization, permanent, non-permanent; use back of page if necessary for additional space)					
II.A.1.b.					
II.A.1.b.i.					
II.A.1.b.ii.					
II.A.1.c.					
II.A.1.c.i.					
II.A.1.c.ii.					
II.A.1.d.					
II.A.1.d.i.					
II.A.1.d.ii.					
II.A.1.e.					
II.A.1.e.i.					
II.A.1.e.ii.					

CONFIDENTIAL

II.A.2. Non-Specialist					
II.A.2.a. Family Med					
II.A.2.a.i. permanent					
II.A.2.a.ii. non-permanent					
II.A.1. Others (please specify / follow format above – specialization, permanent, non-permanent; use back of page if necessary for additional space)					
II.A.1.b.					
II.A.1.b.i.					
II.A.1.b.ii.					
II.A.1.c.					
II.A.1.c.i.					
II.A.1.c.ii.					
II.A.1.d.					
II.A.1.d.i.					
II.A.1.d.ii.					
II.A.1.e.					
II.A.1.e.i.					
II.A.1.e.ii.					
II.B. Nurses					
II.B.1. Permanent					
II.B.2. Non-permanent					
II.C. Nursing Aide					
II.C.1. Permanent					
II.C.2. Non-permanent					
II.D. Psychologist					
II.D.1. Permanent					
II.D.2. Non-permanent					
II.E. Psychometrician					
II.E.1. Permanent					
II.E.2. Non-permanent					
II.F. Physical Therapist					
II.F.1. Permanent					
II.F.2. Non-permanent					

CONFIDENTIAL

II.G. Occupational Therapist					
II.G.1. Permanent					
II.G.2. Non-permanent					
II.H. Social Worker					
II.H.1. Permanent					
II.H.2. Non-permanent					
II.I. Others *(please specify, follow format above, please use back space of the paper)*					

II.J. How do you prioritize personnel for training? *(please rank from 1 being most prioritized to 10 being least prioritized)*

______ II. J. 1. Status of employment (permanent/non-permanent)

______ II.J.2. Position/Designation

______ II.J.3. Length of tenure

______ II.J.4. Age

______ II.J.5. Gender

______ II.J.6. Profession

______ II.J.7. Work performance rating

______ II.J.8. Civil Status

______ II.J.9. Specialty

______ II.J.10. Others *(please add at least one and rank)*

CONFIDENTIAL

III. Health Services

III.A. Clinical Practice<br>*(please check corresponding box)*	YES	NO
III.A.1. Is there a Comprehensive Geriatric Assessment conducted to all older patients (60 years old and above)?		
III.A.2.a. If no, briefly explain why?		
III.A.2. What assessment tools do you use to evaluate older patients?<br>III.A.2.a.<br>III.A.2.b.<br>III.A.2.c.<br>III.A.2.d.		
III.A.3. Does the assessment include the following?		
III.A.3.a. Physical Examination		
III.A.3.b. Vital Signs		
III.A.3.c. Neurologic Evaluation		
III.A.3.d. Mental Evaluation		
III.A.3.e. Cognitive Evaluation (MOCA)		
III.A.3.f. Assessment of Frailty		
III.A.3.g. Medication Reconciliation		
III.A.3.h. Gait Analysis		
III.A.3.i. Strength Evaluation		
III.A.3.j. Balance and Coordination Assessment		
III.A.3.k. Nutritional Evaluation		
III.A.3.l. Assessment of the Quality of life		

CONFIDENTIAL

III.A.5. Is the facility accredited by PhilHealth?		
III.A.5.a. Can older patients avail of the following PhilHealth benefits in the facility? *(check all that applies)*<br>III.A.5.a.1. In-patient Benefits ◯<br>III.A.5.a.2. Out-patient Benefits ◯<br>III.A.5.a.2.i. PCB-1 ◯<br>III.A.5.a.2.ii. EPCB ◯<br>III.A.5.a.3. Z Benefits ◯<br>III.A.5.a.4. Others *(please specify)* ___________________		
III.A.5.b. If yes on Z Benefits, what package can the older patients avail?<br>◯ III.A.5.b.1. Breast Cancer<br>◯ III.A.5.b.2. Prostate Cancer<br>◯ III.A.5.b.3. Cervical Cancer<br>◯ III.A.5.b.4. Colon and Rectum Cancer<br>◯ III.A.5.b.5. ESRD eligible for requiring Kidney transplant<br>◯ III.A.5.b.6. PD First - ESRD requiring Peritoneal Dialysis<br>◯ III.A.5.b.7. CABG surgery<br>◯ III.A.5.b.8. MORPH<br>◯ III.A.5.b.9. Orthopedic Implants<br>III.A.5.b.10. Others *(please specify)* ____________________		
III.A.5.c. If not a PhilHealth accredited facility, please briefly explain why?		
III.A.4. Does the PhilHealth registration of older patients get processed if they are non-PhilHealth members?		
III.A.4.a. If no, briefly explain why?		

CONFIDENTI

III.A.6. Can a senior citizen avail of all the discounts and privileges they are entitled in your facility?		
III.A.6.a. If no, please briefly explain why?		
III.A.7.a. Can a senior citizen avail of the zero balance bill upon discharge from your facility?		
III.A.7.b. If no, briefly explain why?		
III.A.8.a. Is there free Pneumococcal vaccination for senior citizens?		
III.A.8.b. Is there free Influeza vaccination for senior citizens?		
III.A.9.a. If yes, available for? *(check all that applies)*<br>III.A.9.a 1. ALL senior citizens ◯ *(if checked, proceed to next number)*<br>III.A.9.a.2. INDIGENT senior citizens ◯<br>III.A.9.a.3. 60-65 yeas old ◯<br>III.A.9.a.4. Others: *(please specify)* ______________________		
III.A.9.b. If not all, briefly explain why?		

CONFIDENTIAL

III.A.10. Can a senior citizen avail of the following for free?		
III.A.10.a. Xray		
III.A.10.b. CBC		
III.A.10.c. Blood Chemistry		
III.A.10.d. FBS/RBS		
III.A.10.e. Lipid Profile		
III.A.10.f. Urinalysis		
III.A.10.g. Stool Examination		
III.A.10.h. Sputum Examination		
III.A.10.i. Digital Rectal Examination (for males)		
III.A.10.j. Breast and Cervical Examination (for females)		
III.A.10.k. Pap-smear (for females)		
III.A.10.l. ECG		
III.A.10.m. Diagnostic Ultrasound		
III.A.10.n. Bone Density Examination		
III.A.10.o. Others: *(please specify)*______________________________		

III.A.10.k. If not all, briefly explain why?

III.A.11. Are these medicines available in your facility?
(check all that applies)

III.A.11.a. Antihypertensive

- III.A,9.a.1. Losartan ☐
- III.A.9.a.2. Amlodipine ☐

III.A.11.b. Antihyperglycemic

- III.A.9.b.1. Metformin ☐
- III.A.9.b.2. Gliclazide ☐
- III.A.9.b .3. Insulin ☐

III.A.11.c. Antihyperlipidemic

CONFIDENTIAL

III.A.9.c.1. Simvastatin ☐

III.A.11.d. Antiinfectives

III.A.9.d.1. Amoxicillin ☐

III.A.9.d.2. Azythromycin ☐

III.A.9.d.3. Cefuroxime ☐

III.A.9.d.4. Coamoxiclav ☐

III.A.9.d.5. Doxycycline ☐

III.A.11.e. GIT

III.A.9.e.1. Omeprazole ☐

III.A.9.e.2. Loperamide ☐

III.A.11.f. Dementia

III.A.9.f.1. Memantine ☐

III.A.11.g. Anticonvulsants

III.A.9.f.1. Carbamazepine ☐

III.A.9.f.2. Valproic Acid ☐

III.A.11.h. Antipsychotics

III.A.9.g.1. Clozapine ☐

III.A.9.g.2. Quetiapine ☐

III.A.9.g.3. Chlorpromazine ☐

III.A.11.i. Antidepresants

III.A.9.h.1. Fluoxetine ☐

III.A.9.h.2. Sertraline ☐

III.A.11.j. Cancer Medicines

III.A.9.i.1. Carboplatin ☐

III.A.9.i.2. Cisplatin ☐

III.A.9.i.3. Cyclophospamide ☐

III.A.9.i.4. Docetaxyl ☐

III.A.9.i.5. Tamoxifen ☐

CONFIDENTIAL

III.A.11.k. Herbal Medications	
III.A.9.j.1. Lagundi	
III.A.9.j.2. Sambong	

III.A.11.l. Can senior citizens avail all these medicines for free?		
III.A.11.m. If no, briefly explain why?		

III.A.12.a. Is there free counselling and psychosocial services for older patients?		
III.A.12.b. Is there free counselling and psychosocial services for older patients' family?		
III.A.12.b. Is there free counselling and psychosocial services for older patients' care giver?		
III.A.13. Is physical rehabilitation services available for older patients?		
III.A.14. Is palliative care and end of life care services provided for older patients?		
III.A.14.b. Is there training for family members on palliative care?		
III.A.15.a. Are there discharge instructions provided when the patient leaves the hospital?		
III.A.15.b. Are there patients referred to a Health Center/Nursing Home upon discharge?		
III.A.15.c. Is there a protocol for follow-up of discharged patients?		
III.A.15.d. If yes, what do you do with the data of the follow-up program?		
III.A.16. Are free ambulance services available for senior citizens?		

CONFIDENTIAL

III.B. Access	YES	NO
III.B.1. Is there a fast lane for Senior Citizens?		
III.B.2. Are there available health educating activities while in the waiting area or inside the patient rooms such as:		
III.B.2.a. Television that plays health advocacy ads		
III.B.2.b. Reading materials		
III.B.2.c. Health Counselor		
III.B.3. Which of the following health educating activities are available in the waiting area:		
III.B.3.a. wheelchair for patients who are in distress or who are compromised in ambulating		
III.B.3.b. wheeled-bed for bed bound patients		
III.B.4. Are there chairs or space allotted for older patients in the waiting area of the out-patient department?		
III.B.5.a. Is there a comfort room visible from the allotted waiting area of older patients?		
III.B.5.b. Can a senior citizen walk a straight path from the waiting area to the comfort room?		
III.B.6.a. Is there a canteen visible from the allotted waiting area of older patients?		
III.B.6.b. Can a senior citizen walk a straight path from the waiting area to the canteen?		
III.B.6.c. Does the canteen sell food appropriate for the nourishment of older persons?		
III.B.7. Is there a designated parking space for senior citizens?		
III.B.8. Are there ramps and/or lifts?		

CONFIDENTIAL

III.C. Physical Environment	YES	NO
III.C.1.a. Is there a continuous supply of clean water?		
III.C.1.b. Is there a continuous supply of potable drinking water?		
III.C.2. Is there electricity in the facility?		
III.C.2.b. Is there a source of electricity during periods of power interruption?		
III.C.3.a. Are the windows and doors open in non-air-conditioned spaces in the facility to provide ventilation?		
III.C.3.b. Are there electric fans to provide additional ventilation?		
III.C.3.c. Are enclosed spaces in the facility provided with air conditioning for ventilation?		
III.C.4.a. Do the open spaces in the facility have enough day time brightness for older patients to read, and distinguish colors, persons and objects?		
III.C.4.b. Do the lights inside closed spaces in the facility provide enough illumination for older patients to read and distinguish color, objects and persons?		
III.C.4.c. Do the lights during nighttime provide enough illumination for older patients to read and distinguish color, objects and persons?		
III.C.5. Are the pathways in the facility clear of obstruction?		
III.C.5.a. From the facility entrance to the waiting area		
III.C.5.b. From the waiting area to consultation room		
III.C.5.c. From the waiting area to the bathroom		
III.C.5.d. From the waiting area to the laboratory		
III.C.6. Is the floor made of non-slippery material?		
III.C.7. Are the stair and steps well marked with colors that are easily recognizable?		

CONFIDENTIAL

III.C.8. Are the signage readable upon entering the facility?		
III.C.9. Are the trash bins visible and labeled?		
III.C.10. Are no-smoking signs prominently displayed around the facility?		
III.C.11.a. Are offices and working areas labeled with the names of occupants?		
III.C.11.b. Are the labels readable for older patients in the waiting area?		

III.D. Safety and Security	Yes	No
III.D.1. Are there security personnel in the facility?		
III.D.2. Does the staff wear proper uniform?		
III.D.3. Do all personnel wear proper identification cards?		
III.D.3.a. Does this identification card bears the name and designation of the personnel?		
III.D.3.b. Is written information in the identification cards in bold and big letters readable for older patients?		
III.D.4. Do personnel in the facility wear proper protective equipment in handling older patients?		
III.D.5. Are the staff properly trained to assist older patients in times of disaster?		
III.D.6. Are the emergency exits clearly labeled and visible from the waiting area?		
III.D.7. Are the pathways to the emergency exit cleared of obstructions?		

CONFIDENTIAL

III.D. PATIENT CARE AND PARTICIATION	YES	NO
III.D.1. Is accurate information provided to the patient or his/ her surrogate decision maker of all the processes and procedures that he/she will undergo such as:		
III.D.1.a. Patient status		
III.D.1.b. Treatment goals		
III.D.1.c. Plan of Care		
III.D.1.d. Available services, procedures and cost		
III.D.1.e. Alternative source of care and services when not available in the facility		
III.D.2. Is proper written consent of the patient or his/her surrogate decision maker acquired prior to conducting any procedure or treatment?		
III.D.3.a. Are all processes and procedures documented?		
III.D.3.b. Are patient files and records on documented processes and procedures properly kept?		
III.D.3.c. Are these files available for the patient if he/she wishes to see it?		
III.D.3.d. Are these files kept confidential from unauthorized access?		
III.D.4. Is quality of life of the patient assessed during the interviews?		
III.D.5. Is there a mechanism for the patient to provide feedback, suggestions, comments or recommendations to the facility?<br>III.D.5.a. What are these? (*check all that may apply)*<br>Suggestion box _______<br>Evaluation form _______<br>Private letter _______<br>Others please specify: __________________________		

CONFIDENTIAL

I. Health Financing

II.

Data pertains to fiscal year 2018

IV.A. What is the total operating budget of the hospital?	Php
IV.B. Do you have a data on the budget used for Older patients?	IV.B.1. Yes ◯ IV.B.2. No ◯
IV.B.1. If yes, what is the budget for older patients?	Php
IV.B.2. If no, please briefly explain why?	
IV.C. Do you adhere to the no balance billing policy of PhilHelath for senior citizens?	IV.C.1. Yes ◯ IV.C.2. No ◯
IV.D. Do you have data of the total amount reimbursed by PhilHealth in your institution for senior citizens?	IV.D.1. Yes ◯ IV.D.2. No ◯
IV.D.1. If yes, what is the total reimbursed amount for older patients?	Php
IV.D.2. If no, please briefly explain why?	
IV.E. What is the average monthly amount of PhilHealth reimbursement?	Php

CONFIDENTIAL

IV. Information System

	Yes	No
V.A. Does the facility maintain medical records of patients?		
V.B. Does the facility have a data on top causes of mortality and morbidity at the hospital?		
V.C. Does the facility have a registry of diseases of older Filipinos?		
V.C.1. Does it include:		
V.C.1.a. Hypertension		
V.C.1.b. Diabetes		
V.C.1.c. Cardiovascular Diseases		
V.C.1.c.i. Stroke or Cerebrovascular Attack		
V.C.1.c.ii. Heart Attack or Myocardial Infarction		
V.C.1.d. Respiratory Tract Diseases		
V.C.1.d.i. Pulmonary Tuberculosis		
V.C.1.d.ii. Asthma		
V.C.1.d.iii. COPD		
Others *(please specify)*		
V.C.1.e. Cancer		
V.C.1.e.i. Prostate		
V.C.1.e.ii. Lung		
V.C.1.e.iii. Cervical		
V.C.1.e.iv. Breast		
V.C.1.e.v. Colon		
V.C.1.e.vi. Others *(please specify)*		

CONFIDENTIAL

V.C.1.f. Mental Disorders		
V.C.1.f.i. Dementia		
V.C.1.f.ii. Depression		
V.C.1.f.vi. Others *(please specify)*		
V.D. Does the facility have a registry of older persons with disability?		
V.D.1. Visual		
V.D.2. Communication		
V.D.3. Orthopedic		
V.D.4. Learning		
V.D.5. Mental		
V.D.6. Psychosocial		
V.D.7. Chronic Illness		
V.D.8. Neurologic (seizures, convulsions)		

V.E. Is there an online or web based database of patient records?		
V.E.1. If yes, do you update data regularly?		
(check all that apply)		
V.E.1.a. iHomis		
V.E.1.b. UDRS		
V.E.1.c. Others *(please specify)*		
V.E.2. If no, what are the reasons? *(check all that apply)*		
V.E.2.a. No Computer		
V.E.2.b. No or unstable internet access		
V.E.2.c. No trained personnel		
V.E.2.d. Others *(please specify)*		

CONFIDENTIAL

V. Health Policy

	Yes	No
VI.A. IS there a policy and program for the care of older persons?		
VI.B. Is there a policy and program regarding patients with dementia?		
VI.C. Does the facility have a policy and program regarding palliative care?		
VI.D. Is there a policy regarding pain management for older patients?		
VI.E. Is there a policy on how to handle cases of neglect and abuse of older patients?		
VI.F. Is there a policy on how to manage older patients during times of calamities?		
VI.G. Is tehre a guideline on referring, transferring and monitoring older patients?		
VI.H. Is there a policy on the rules and regulations of the institution?		
VI.I. Is there a policy regarding managing patients' records?		

CONFIDENTIAL

---E N D---

FITforFRAIL

Review of Health Services

Primary Health Center

Name of Facility: ____

I. Facility Demographics

	Yes	No
I.A. Total Population of Municipality (December 2018)		
I.B. Population of 60 years old and above (December 2018)		
I.C. Total number of patients (January 2018 - December 2018)		
I.D. Average patients per day		
I.E. Total number of patients age 60 and above (January 2018 – December 2018)		
I.F. Average patients of 60 year old and above per day		
I.G. Do you have a health program for senior citizens?		
I.H. What mechanism is in place to evaluate the progress of the program?		

I.H.1. Scheduled progress report	
I.H.2. Program implementation review	
I.H.3. Client feedback survey	
I.H.4. Regular Meetings	
I.H.5. Special Audits	
I.H.6. Others *(please specify)*	

CONFIDENTIAL

II. Human Resource, Competencies and Training

Workforce	Total Number	Competencies and Trainings		
		**Formal**	**Informal**	**Trainings**
		(Completed Specialization or Post Graduate degree in Geriatrics or Gerontology)	*(Certificate of Training in the Conduct of Geriatric Assessment and Management)*	*(Other trainings related to the care of the Senior Citizens)*
II.A. Doctor of Medicine				
II.A.1. Permanent/Plantilla				
II.A.2. Casual				
II.A.3. Contractual/Job Hire/Project				
II.A.4. HRH (DTTB)				
II.A.5. Others				
II.B. Nurses				
II.B.1. Permanent/Plantilla				
II.B.2. Casual				
II.B.3 Contractual/Job Hire/Project				
II.B.4. HRH NDP				
II.B.5. Others				
II.C. Nursing Assistant/Attendant				
II.C.1. Permanent/Plantilla				
II.C.2. Casual				
II.C.3. Contractual/Job Hire/Project				
II.C.4. HRH (DTTB)				
II.C.5. Others				
II.D. Midwives				
II.D.1. Permanent/Plantilla				
II.D.2. Casual				
II.D.3. Contractual/Job Hire/Project				
II.D.4. HRH (DTTB)				
II.D.5. Others				

CONFIDENTIAL

II.E. Dentists				
II.E.1. Permanent/Plantilla				
II.E.2. Casual				
II.E.3. Contractual/Job Hire/Project				
II.E.4. HRH (DTTB)				
II.E.5. Others				
II.F. Lab Technicians / MedTech				
II.F.1. Permanent/Plantilla				
II.F.2. Casual				
II.F.3. Contractual/Job Hire/Project				
II.F.4. HRH (DTTB)				
II.F.5. Others				
II.G. Nutritionist/Dietician				
II.G.1. Permanent/Plantilla				
II.G.2. Casual				
II.G.3. Contractual/Job Hire/Project				
II.G.4. HRH (DTTB)				
II.G.5. Others				
II.H. Others *(please follow format)*				

CONFIDENTIAL

II.J. How are personnel prioritized for training? *(please rank from 1 being most prioritized to 10 being least prioritized)*

_____ II.J.1. Status of employment (permanent/non-permanent)

_____ II.J.2. Position/Designation

_____ II.J.3. Length of tenure

_____ II.J.4. Age

_____ II.J.5. Profession

_____ II.J.6. Work performance rating

_____ II.J.7. Civil Status

_____ II.J.8. Gender

_____ II.J.9. Specialty

_____ II.J.10. Others *(please add at least one and rank)*

CONFIDENTIAL

III. Health Services

III.A. Clinical Practice<br>*(please check corresponding box)*	YES	NO
III.A.1. Is there free medical and dental check-up for senior citizens?		
III.A.2. Is there a brief Geriatric Assessment to older patients (60 years old and above)?		
III.A.2.a. If no, briefly explain why?		
III.A.3. What assessment tools do you use to evaluate older patients?<br>III.A.3.a.<br>III.A.3.b.<br>III.A.3.c.<br>III.A.3.d.		
III.A.4. Does the assessment include the following procedures?		
III.A.4.a. Physical Examination		
III.A.4.b. Vital Signs		
III.A.4.c. Neurologic Evaluation		
III.A.4.d. Mental Evaluation		
III.A.4.e. Cognitive Evaluation (MOCA)		
III.A.4.f. Assessment of Frailty		
III.A.4.g. Medication Reconciliation		
III.A.4.h. Gait Analysis		
III.A.4.i. Strength Evaluation		
III.A.4.j. Balance and Coordination		
III.A.4.k. Nutritional Evaluation		
III.A.5. Is the facility accredited by PhlHealth?		
III.A.5.a. What type of accreditation? *(check all that apply)*<br>III.A.5.a.i. PCB-1 ◯<br>III.A.5.a.ii. EPCB ◯<br>III.A.5.a.iii. Others please specify__________________		

CONFIDENTI

III.A.5.b. If no, briefly explain why?		
III.A.6.a. Does the PhilHealth registration of older patients gets processed if they are non-PhilHealth members?		
III.A.6.b. If no, briefly explain why?		
III.A.7.a. Is there free Pneumococcal vaccination for senior citizens?		
III.A.7.b. Is there free Influeza vaccination for senior citizens?		
III.A.7.c. Available for? *(check all that applies)*<br>III.A.7.c.1. ALL senior citizens ◯ *(if yes, proceed to next number)*<br>III.A.7.c.2. INDIGENT senior citizens ◯<br>III.A.7.c.3. 60-65 yeas old ◯<br>III.A.7.c.4. Others: *(please specify)* ______________________		
III.A.7.d. If not all, briefly explain why?		
III.A.8. Can a senior citizen avail of the following for free?		
III.A.8.a. Chest Xray		
III.A.8.b. CBC		
III.A.8.c. FBS/RBS		
III.A.8.d. Lipid Profile		
III.A.8.e. Urinalysis		
III.A.8.f. Stool Examination		
III.A.8.g. Sputum Examination		
III.A.8.h. Digital Rectal Examination (for males)		
III.A.8.i. Breast and Cervical Examination (for females)		
III.A.8.j. Others: *(please specify)*________________________		

CONFIDENTIAL

III.A.8.k. If not all, briefly explain why?

III.A.9. Are these medicines available in your facility?
(check all that applies)

III.A.9.a. Antihypertensive

- lll.A,9.a.1. Losartan ☐
- lll.A.9.a.2. Amlodipine ☐

III.A.9.b. Antihyperglycemic

- III.A.9.b.1. Metformin ☐
- III.A.9.b.2. Gliclazide ☐
- III.A.9.b .3. Insulin ☐

III.A.9.c. Antihyperlipidemic

- III.A.9.c.1. Simvastatin ☐

III.A.9.d. Antiinfectives

- III.A.9.d.1. Amoxicillin ☐
- III.A.9.d.2. Azythromycin ☐
- III.A.9.d.3. Cefuroxime ☐
- III.A.9.d.4. Coamoxiclav ☐
- III.A.9.d.5. Doxycycline ☐

III.A.9.e. GIT

- III.A.9.e.1. Omeprazole ☐
- III.A.9.e.2. Loperamide ☐

III.A.9.f. Dementia

- III.A.9.f.1. Memantine ☐

III.A.9.g. Anticonvulsants

- III.A.9.f.1. Carbamazepine ☐
- III.A.9.f.2. Valproic Acid ☐

CONFIDENTIAL

III.A.9.h. Antipsychotics	
III.A.9.g.1. Clozapine	
III.A.9.g.2. Quetiapine	
III.A.9.g.3. Chlorpromazine	
III.A.9.i. Antidepresants	
III.A.9.h.1. Fluoxetine	
III.A.9.h.2. Sertraline	
III.A.9.j. Cancer Medicines	
III.A.9.i.1. Carboplatin	
III.A.9.i.2. Cisplatin	
III.A.9.i.3. Cyclophospamide	
III.A.9.i.4. Docetaxyl	
III.A.9.i.5. Tamoxifen	
III.A.9.k. Herbal Medications	
III.A.9.j.1. Lagundi	
III.A.9.j.2. Sambong	

III.A.9.l. Can senior citizens avail of all these medicines for free?		
III.A.9.m. If no, briefly explain why?		

CONFIDENTIAL

III.A.10.a. Is there free counselling and psychosocial support services to older patients?		
III.A.10.b. Is there free counselling and psychosocial support services to older patients' families?		
III.A.10.c. Is there free counselling and psychosocial support services to caregivers of older patients??		
III.A.11. Do you refer patients to a higher-level facility if the assessed signs and symptoms needs further evaluation or for an advanced and/or specialized type of care?		
III.A.11.a. How far is the nearest referral institution from this facility? _____________Kms.<br>III.A.11.b. How long does it take to get there from this facility? ____________________(Min. / Hrs.)<br>III.A.11.c. What hospital classification is this institution?<br>Level 1______<br>Level 2______<br>Level 3______		
III.A.12. Are there older patients referred by a higher-level facility for follow up and monitoring?		
III.A.13. Are there scheduled community visits to follow up and monitor patients?		
III.A.14.a. Is physical rehabilitation services available for older patients?		
III.A.14.b. Can a senior citizen avail physical rehabilitation services for free?		
III.A.14.c. If no, please briefly explain why?		
III.A.15. Is there a short term infirmary care service?		
III.A.16. Is there an emergency basic life support service?		
III.A.17. Are free ambulance services available for senior citizens?		

CONFIDENTIAL

III.A.18.a. Is palliative care and end of life care services provided for older patients?		
III.A.18.b. Is there training for family members for palliative care?		

III.B. Access	YES	NO
III.B.1. Is there a fast lane for Senior Citizens?		
III.B.2. Which of the following health education activities are available in the waiting area:		
III.B.2.a. Television that plays health advocacy ads		
III.B.2.b. Reading materials readable by older patients		
III.B.2.c. Health counselor giving health talks		
III.B.3. What are the available mobility assistive devices in the facility?:		
III.B.3.a. wheelchair for patients who are in distress or who are compromised in ambulating		
III.B.3.b. wheeled-bed for bed bound patients		
III.B.4. Are there chairs or space allotted specifically for older patients in the waiting area?		
III.B.5.a. Is there a comfort room visible from the allotted waiting area of older patients?		
III.B.5.b. Can a senior citizen walk a straight path from the waiting area to the comfort room?		
III.B.6.a. Is there a canteen visible from the allotted waiting area of older patients?		
III.B.6.b. Can a patient walk a straight path from the waiting area to the canteen?		
III.B.6.c. Does the canteen sell food appropriate for the nourishment of older persons?		
III.B.7. Is there a designated parking space for senior citizens?		
III.B.8. Are there ramps and/or lifts?		

CONFIDENTIAL

III.C. Physical Environment	YES	NO
III.C.1.a. Is there a continuous supply of clean water?		
III.C.1.b. Is there a continuous supply of potable drinking water?		
III.C.2. Is there electricity in the facility?		
III.C.2.b. Is there a source of electricity during periods of power interruption?		
III.C.3.a. Are the windows and doors open on non-air-conditioned spaces in the facility to provide ventilation?		
III.C.3.b. Are there electric fans to provide additional ventilation?		
III.C.3.c. Are enclosed spaces in the facility provided with air conditioning for ventilation?		
III.C.4.a. Do the open spaces in the facility have enough day time brightness for older patients to read, and distinguish colors, persons and objects?		
III.C.4.b. Do the lights inside closed spaces in the facility provide enough illumination for older patients to read and distinguish color, objects and persons?		
III.C.4.c. Do the lights during nighttime provide enough illumination for older patients to read and distinguish color, objects and persons?		

III.C.5. Are the pathways in the facility clear of obstruction?		
III.C.5.a. From the facility entrance to the waiting area		
III.C.5.b. From the waiting area to consultation room		
III.C.5.c. From the waiting area to the bathroom		
III.C.5.d. From the waiting area to the laboratory		
III.C.6. Is the floor made of non-slippery material?		
III.C.7. Are the stair and steps well marked with colors that are easily recognizable?		

CONFIDENTIAL

III.C.8. Are the signage readable upon entering the facility?		
III.C.9. Are the trash bins visible and labeled?		
III.C.10. Are no-smoking signs prominently displayed in the facility??		
III.C.11.a. Are offices and working areas labeled with the names of occupants?<br><br>III.C.11.b. Are the labels readable for older patients in the waiting area?		

III.D. Safety and Security	Yes	No
III.D.1. Are there security personnel in the facility?		
III.D.2. Does the staff wear proper uniform?		
III.D.3. Do all personnel wear proper identification cards?		
III.D.3.a. Does this identification card bear the name and designation of the personnel?		
III.D.3.b. Is written information in the identification cards in bold and big letters readable for older patients?		
III.D.4. Do the personnel in the facility wear proper protective equipment in handling older patients?		
III.D.5. Are the staff properly trained to assist older patients in times of disaster?		
III.D.6. Are the emergency exits clearly labeled and visible from the waiting area?		
III.D.7. Are the pathways to the emergency exit cleared of obstructions?		

CONFIDENTIAL

III.D. PATIENT CARE AND PARTICIPATION	YES	NO
III.D.1. Is accurate information and knowledge provided to the patient or his/ her surrogate decision maker of all the processes and procedures that he/she will undergo such as:		
III.D.1.a. Patient status		
III.D.1.b. Treatment goals		
III.D.1.c. Plan of Care		
III.D.1.d. Available services, procedures and cost		
III.D.1.e. Alternative source of care and services when not available in the facility		
III.D.2. Is written consent of the patient or his/her surrogate decision maker acquired prior to conducting any procedure or treatment?		
III.D.3.a. Are all processes and procedures documented?		
III.D.3.b. Are patient files and records on documented processes and procedures properly kept?		
III.D.3.c. Are these files available for the patient if he/she wishes to see it?		
III.D.3.d. Are these files kept confidential from unauthorized access?		
III.D.4. Is quality of life of the patient assessed during the interviews?		
III.D.5. Is there a mechanism for the patient to provide feedbacks, suggestions, comments or recommendations to the facility?		
III.D.5.a. What are these? (*check all that may apply)*<br>III.D.5.a.1. Suggestion box _______<br>III.D.5.a.2. Evaluation form _______<br>III.D.5.a.3. Private letter _______<br>III.D.5.a.4. Others please specify: _________________________		

CONFIDENTIAL

IV. Health Financing

Data pertains to fiscal year 2018

	Php
IV.A. What is the total budget of the Municipality/City?	
IV.B. What is the total budget for health of the Municipality/City?	
IV.C. What is the total budget of the RHU?	
IV.D. What is the budget allotted for senior citizens?	
IV.E. What is the total budget allotted for the health of senior citizens?	
IV.F. What is the total cost of vaccines purchased by the RHU for senior citizens?	
IV.G. What is the total cost of medicines purchased by the RHU for senior citizens?	
IV.H. What is the total amount of funds generated from other sources (donations, projects, etc)	
IV.I. What is the average amount that the PhilHealth reimburses the facility every month?	

CONFIDENTIAL

V. Information System

	Yes	No
V.A. Does the facility maintain medical records of patients?		
V.B. Does the facility have a data on top causes of mortality and morbidity at the municipality/city?		
V.C. Does the facility have a registry of diseases of older Filipinos?		
V.C.1. Does it include:		
V.C.1.a. Hypertension		
V.C.1.b. Diabetes		
V.C.1.c. Cardiovascular Diseases		
V.C.1.c.i. Stroke or cerebrovascular Attack		
V.C.1.c.ii. Heart attack or myocardial Infarction		
V.C.1.d. Respiratory Tract Diseases		
V.C.1.d.i. Pulmonary Tuberculosis		
V.C.1.d.ii. Asthma		
V.C.1.d.iii. COPD		
Others *(please specify________________________)*		
V.C.1.e. Cancer		
V.C.1.e.i. Prostate		
V.C.1.e.ii. Lung		
V.C.1.e.iii. Cervical		
V.C.1.e.iv. Breast		
V.C.1.e.v. Colon		
V.C.1.e.vi. Others *(please specify)*		

CONFIDENTIAL

V.C.1.f. Mental Disorders		
V.C.1.f.i. Dementia		
V.C.1.f.ii. Depression		
V.C.1.f.vi. Others *(please specify)*		
V.D. Does the facility have a registry of older persons with disability?		
V.D.1. Visual		
V.D.2. Communication		
V.D.2.a. Auditory		
V.D.2.b. Speech and Language		
V.D.3. Orthopedic		
V.D.4. Learning		
V.D.5. Mental		
V.D.6. Psychosocial		
V.D.7. Chronic Illness		
V.D.8. Neurologic (seizures, convulsions)		

V.E. Is there an online or web based database of patient records?		
V.E.1. If yes, do you update data regularly? *(check all that apply)*		
V.E.1.a. iClinicSys		
V.E.1.b. UDRS		
V.E.1.c. Others *(please specify)*_________________		
V.E.2. If no, what are the reasons? *(check all that apply)*		
V.E.2.a. No Computer		
V.E.2.b. No or unstable internet access		
V.E.2.c. No trained personnel		
V.E.2.d. Others *(please specify)*_________________		

CONFIDENTIAL

VI. Health Policy

	Yes	No
VI.A. Is there a policy and program for the care of older persons?		
VI.B. Is there a policy and program regarding patients with dementia?		
VI.C. Does the facility have a policy and program regarding palliative care?		
VI.D. Is there a policy regarding pain management for older patients?		
VI.E. Is there a policy on how to handle cases of neglect and abuse of older patients?		
VI.F. Is there a policy on how to manage older patients during times of calamities?		
VI.G. Is there a guideline on referring, transferring and monitoring older patients?		
VI.H. Is there a policy on the rules and regulations of the institution?		
VI.I. Is there a policy regarding managing patients' records?		

CONFIDENTIAL

---E N D---
